# Overcoming the “burdens” of overweight: coping strategies for weight stigmatising experiences and influences on psychological outcomes

**DOI:** 10.1186/2050-2974-3-S1-P7

**Published:** 2015-11-23

**Authors:** Silvia Violante-Cumpa

**Affiliations:** 1 Swinburne University of Technology, Melbourne, Australia

## 

Despite some discussion of the effect of weight stigma on psychological outcomes, little is known about specific coping factors that can reduce the impact of stigmatisation among overweight and obese persons. Furthermore, analysis of coping strategies for weight stigma within a stress-coping framework is underdeveloped. This research examined the relationship between weight stigmatising experiences, internalisation, coping strategies, and possible psychological consequences (e.g. depression, anxiety, stress, and self-esteem) through the administration of online questionnaires with qualitative and quantitative measures to overweight and obese persons in the general population.

